# High range of motion in the first ten postoperative days after TKA does not predict superior outcome in the long run

**DOI:** 10.1007/s00402-021-03869-1

**Published:** 2021-03-25

**Authors:** Paul Köglberger, Alexander Wurm, Débora Coraça-Huber, Martin Krismer, Wilhelm Oberaigner, Michael Liebensteiner

**Affiliations:** 1 Medical University of Innsbruck, Innsbruck, Austria; 2 Department of Orthopaedics and Traumatology, Medical University of Innsbruck, Innsbruck, Austria; 3 Department of Experimental Orthopaedics, Medical University of Innsbruck, Innsbruck, Austria; 4 Department of Clinical Epidemiology, Tyrolean Federal Institute for Integrated Care, Tirol Kliniken GmbH, Innsbruck, Austria

**Keywords:** Total knee arthroplasty, Range of motion, Fast track, Rapid recovery, Knee replacement

## Abstract

**Introduction:**

To retrospectively investigate the early postoperative range of motion (ROM) (days 4, 7, 10) after total knee arthroplasty (TKA) and to test for associations (a) with long-term outcome in terms of ROM and (b) with a disease-specific knee score.

**Materials and methods:**

A retrospective analysis was performed in patients with previous primary TKA. Data taken from the medical records were ROM from preoperative and postoperative days 4, 7 and 10 and 1 year. As patient-reported outcome the Western Ontario and McMaster Universities Osteoarthritis Index (WOMAC Score) was taken from preoperative and one year after TKA.

**Results:**

316 patients (330 knees) were available. Only negligible correlations were determined between ROM at twelve months postoperative and ROM in the early postoperative days (days 4, 7, 10). Similarly, only negligible correlations were determined between ROM in the early postoperative days (days 4, 7, 10) and the 1-year WOMAC.

**Conclusion:**

From the main findings it would seem that steepness of ROM ascent in the early postoperative days is of minor importance for (a) long-term ROM and (b) long-term knee score outcome after TKA.

## Introduction

In daily clinical practice the question often arises as to when to discharge a patient after routine total knee arthroplasty (TKA). The fact that there is no generally accepted consensus on this question is also reflected by the length of hospital stay, which varies in general between two and 15 days [1–13] with even longer stays observed [14, 15]. Besides other clinical Landmarks like wound drainage or pain control also the progression of early postop range of motion (ROM) may be considered [4, 7, 8, 13, 16]. With regard to ROM it might particularly be speculated whether early postoperative ROM (postoperative day 1–4) is a good indicator of long-term postoperative ROM. Moreover, it might also be speculated whether early postoperative ROM is associated with general outcome after TKA. In such a case it would be useful to accelerate the gain in early ROM immediately after TKA, as seen in many fast-track programs following TKA. Moreover, it would also be justified to use ‚early ROM’ for the decision on when and where to discharge a patient from hospital as well as for identifying those at high risk for poor long-term outcome after TKA.

Regarding associations between early postoperative ROM and long-term ROM after TKA, Bade et al. found that knee flexion at postoperative day 2 was not related to knee flexion at six months postoperative [3]. Others found that greater knee flexion at discharge did not predict knee flexion one year postoperative [17]. Regarding associations between early post-op ROM and long-term knee score outcome after TKA, previous authors reported that the amount of knee flexion (and extension) at discharge after TKA was not associated with the Oxford knee score at one year postoperative [17]. Aside from the above-mentioned two articles, the available evidence is rare.

Based on the above-mentioned lack of evidence it was the aim of the study to test whether early postop ROM (days 4, 7, 10) was associated with ROM at 12 months postoperative (Hypothesis 1) or with the WOMAC Score at 12 months postoperative (Hypothesis 2).

## Materials and methods

The study design was retrospective observational. Data already available from clinical routine were analyzed after approval by the ethics committee of the Medical University. Consecutive patients who previously underwent primary TKA as part of the clinical routine were analyzed. Cases were excluded in the case of: (a) incomplete WOMAC data, (b) incomplete ROM data, (c) primary prostheses other than cruciate-retaining. Included were patients with Triathlon CR, Scorpio CR and Scorpio NRG CR implants (Stryker, Kalamazoo, MI, USA).

During the study period (January 2005 to August 2015), 2093 TKA were performed at our institution. Of those, 988 had to be excluded because either the preoperative or postoperative or both WOMAC Scores were incomplete. Of the remaining 1105 TKA 242 were excluded because other implants than above-mentioned CR implants had been used. Of the remaining 863 primary TKA only 330 cases could be included in the final analysis. The rest had to be excluded because no information on ROM was available.

ROM data collected with goniometers were taken from the medical records for the following points in time: preoperative, postoperative days 4, 7 and 10 and 1 year (± 6 months) postoperative. The early postoperative measurements were taken from the obligatory daily ROM documentation of our physiotherapists. The one-year ROM was taken from the documentation of the one-year outpatient visit.

For patient-reported outcome measurement the Western Ontario and McMaster Universities Osteoarthritis Index (WOMAC) was applied in the German-language version [18]. The questionnaire was completed shortly before surgery during the hospital stay (preoperatively) and postoperatively the questionnaire was sent directly to the patient one year after surgery. The WOMAC questionnaire collects data on pain, stiffness, and physical function. Every item was completed on an 11-point scale and converted for analysis purposes to a scale from 0 to 100, 0 denoting the best and 100 the worst response. The score for each of the three main dimensions is defined as the sum of all item scores divided by the number of items. The total score was defined as the sum of pain, stiffness and function scores divided by three.

Data were extracted from the records and stored in spreadsheet format. Further analyses were performed with SPSS. As descriptive statistics medians and inter-quartile-ranges were determined. Kolmogorov–Smirnov tests indicated that the data sets were not normally distributed. Therefore, the above-mentioned Hypotheses 1–4 were tested with Spearman correlation coefficients; alpha was defined as 0.05. With regard to Spearman's correlation coefficient, the size of the correlation was assumed as very high from 0.90 to 1.00, high from 0.70 to 0.90, moderate from 0.50 to 0.70, low from 0.30 to 0.50 and negligible from 0.00 to 0.30 [19].

## Results

316 patients (330 knees; 140 left, 190 right) with a median age of 70 years (IQR 12) were available for analysis.

Knee ROM (Medians ± Interquartile-Ranges) changed from 110 ± 20° preoperative to 65 ± 20° on day 4, further to 85 ± 15° on day 7 and 90 ± 15° on day 10. Twelve months after surgery ROM was 110 ± 20°. The total WOMAC Score was 52 ± 28 preoperative and changed to 16 ± 29 at 12 months postoperative (Table 1).

**Table 1:** Descriptive statistics of ROM and knee score data

	Median	IQR
ROM [°]		
Preoperative	110	20
Day 4	65	20
Day 7	85	15
Day 10	90	15
12 months	110	20
WOMAC total		
Preoperative	52	28
12 months	16	29
WOMAC pain		
Preoperative	50	30
12 months	12	28
WOMAC stiffness		
Preoperative	55	40
12 months	20	30
WOMAC function		
Preoperative	54	29
12 months	16	32

Regarding Hypothesis 1, negligible correlations were determined between ROM at twelve months postoperative and ROM in the early postoperative days (days 4, 7, 10) (Table 2).

**Table 2:** Correlation analysis between ROM after 12 months postoperative and in the early postoperative days

	ROM 12 months
	coefficient^a^
	*p* value
ROM	
Day 4	0.105
	0.097
Day 7	0.219
	0.000*
Day 10	0.243
	0.001*

^a^Spearman’s correlation coefficient

*Significant correlation at the 0.05 level (2-tailed)

Similarly, regarding Hypothesis 2, only negligible correlations were determined between ROM in the early postoperative days (days 4, 7, 10) and the 12-month WOMAC outcome (Table 3).

**Table 3:** Correlation analysis between ROM in the early postoperative days and the 12-month WOMAC score outcome

	WOMAC total 12 months	WOMAC pain 12 months	WOMAC stiffness 12 months	WOMAC function 12 months
	Coefficienta	Coefficienta	Coefficienta	Coefficienta
	*p* value	*p* value	*p* value	*p* value
*ROM*				
Day 4	− 0.038	− 0.031	− 0.044	− 0.039
	0.501	0.590	0 .434	0.489
Day 7	− 0.097	− 0.059	− 0.112	− 0.107
	0.086	0.295	0.048*	0.058
Day 10	− 0.073	− 0.040	− 0.110	− 0.066
	0.255	0.534	0.086	0.310

^a^Spearman’s correlation coefficient

*Significant correlation at the 0.05 level (2-tailed)

## Discussion

The most important finding made in the study was that the ROM obtained in the early postoperative days (days 4, 7, 10) showed only negligible correlations with the WOMAC score at 12 months postoperative and ROM at 12 months postoperative.

When attempting to compare our findings with previous research, it became obvious that evidence on this specific issue is scarce. Regarding the influence of early postoperative ROM on long-term postoperative ROM, supporting evidence comes from Bade et al., who found that knee flexion at postoperative day 2 did not influence knee flexion at six months postoperative [3]. Our findings are also in good agreement with those obtained by Naylor et al. who reported that knee flexion at discharge did not influence the amount of knee flexion at one year postoperative [17]. However, in contrast to the 330 cases in our study, the above-mentioned previous studies presented 64 [3] and 133 cases [17].

With regard to the influence of early postoperative ROM on long-term postoperative knee score outcome, previous researchers tested for associations between knee flexion at discharge and the Oxford knee score one year postoperative [17]. They found that neither maximum knee flexion nor maximum knee extension at discharge had an influence on the Oxford knee score one year postoperative. This is in good agreement with the findings made in our study. We were not able to find other publications dealing with the above-mentioned specific research question.

Our findings are regarded as highly clinically relevant. Due to the fact that early postoperative ROM did not predict long-term knee score outcome or long-term ROM after TKA, it does not seem necessary to force ROM gain too strongly in the initial postoperative days. Probably, preoperative ROM and proper biomechanics of the TKA are predictors of much greater importance than is steepness of ROM ascent in the early postoperative period. Fast-track rehabilitation programs that aim to substantially accelerate ROM gain in the early postoperative period might also be questioned. Although the findings from the current study support the practice of not forcing ROM gain too aggressively in first postoperative weeks there should be a limit to this in patients that do not achieve a minimum of 90° knee flexion within the first postoperative weeks. Yao et al. demonstrated that these patients can successfully be treated with early manipulating under anesthesia. [20]

Other researchers investigated the effect of *pre*operative ROM instead early-postoperative ROM on long-term ROM outcome. The preoperative maximum knee flexion angle was found positively correlated with the flexion angles at 6 months [3, 5, 21, 22] and 12 months [9, 17, 22–24]. Beside the influence of many other variables on the postoperative range of flexion, greater preoperative flexion angle was considered to have the most positive effect on the long-term flexion achieved after surgery and therefore the preoperative flexion was considered to be the primary determining variable of the long-term flexion outcome after TKA [22, 24]. Others measured preoperative ROM instead the flexion angle and reported that the preoperative ROM was significantly correlated with ROM at 6 months [25] and 12 months [25, 26]. When counting both studies reporting ROM and maximum knee flexion angle there seems to be a strong evidence for the relationship between preoperative and long-term postoperative ROM.

Trying to find publications on a potential relationship between *pre*operative ROM and long-term postoperative knee scores, it appears that there are only few studies available. Naylor et al. reported that the preoperative maximum knee flexion angle was not a significant predictor of the Oxford Knee Score at 1-year postoperative [17]. Similarly, the preoperative maximum knee extension angle did not significantly predict the Oxford Knee Score at 1-year postoperatively [17]. However, others found that preoperative ROM was positively correlated with the long-term Hospital for Special Surgery Score after TKA [27]. In synopsis of these findings, the evidence seems to be rare and conflicting.

The following limitations are acknowledged. This was a retrospective study with the weaknesses typically associated with such studies. We were not able to investigate for parameters other than those already available (WOMAC Score, ROM). It is regarded as significant limitation of the study that from 2093 TKA in the study period only 330 cases could be included in the analysis due to missing WOMAC or ROM data or due to the fact that other than the above-mentioned CR implants had been used. A fact which may have led to selection bias. Another weakness was that ROM was not always measured by the same investigator. It was measured by physical therapists in the early postop phase and by physicians at the 1-year outpatient visit.

Its strength is that the current work generated scientific findings in a field where publications are very rare. Moreover, the findings obtained from the current work are based on case numbers far superior to those of previous research.

## Conclusions

From the main findings that early postoperative ROM did not influence long-term knee score outcome or long-term ROM after TKA, it does not seem necessary to force ROM gain too strongly in the initial postoperative days. Probably, preoperative ROM and proper biomechanics of the TKA are predictors of much greater importance than is steepness of ROM ascent in the early postoperative period.

## Abbreviations

ROM: Postoperative range of motion
SPSS: Statistical Package for the Social Sciences
TKA: Total knee arthroplasty
WOMAC Score: Western Ontario and McMaster Universities Osteoarthritis Index Score
